# The Work of St. Thomas's in Lambeth, by Mr. G. Q. Roberts

**Published:** 1918-12-07

**Authors:** 


### THE WORK OF ST. THOMAS'S IN LAMBETH, by Mr. G. Q. ROBERTS.

Mr. Roberts, whose lecture was made doubly interesting by the exhibition of a large number of slides, first sketched the hospital's history from its establishment as a separate ecclesiastical institution in 1215, under the ægis of Peter de Rupibus, Bishop of Winchester, who later became the well-known Minister of Henry III. His action was to unite under one administration the almonries which had been attached to the old Priory of St. Mary Overy and the Priory of Bermondsey, which priory had been first established by Aylwin Child in 1080. Slides were shown indicating the position of the original hospital on the old Southwark approach to the southern end of London Bridge. In 1507 the hospital buildings had become old and dilapidated, and a fresh site was purchased to the north of St. Thomas's Church, where buildings for the accommodation of the sick poor were erected. The total outlay on site and buildings at that time was £311; it was the same land which, later, was bought by the railway company for nearly a hundred times the figure.

"The King's Hospital."

After the dissolution of the religious houses by Henry VIII. in 1538, the buildings fell into a neglected condition, until, in 1551, the Mayor and Corporation approached Edward VI., and, with the valuable assistance of Bishop Ridley, secured from His Majesty the restoration of the endowment of the ancient hospital. It was first opened as the King's Hospital, but by 1553 the name had been changed back to "St. Thomas's," in memory of Thomas à Becket, the Martyr of Canterbury. By the Charter of 1553, the "Royal Hospitals"—St. Bartholomew's, Bridewell, Christ's Hospital, and St. Thomas's—were united and placed under the control of the Lord Mayor and Corporation of the City of London. At first the administration of Christ's and St. Thomas's was under one head, but after a short interval the details of administration were separated, and Sir Wm. Chester, afterwards Lord Mayor of London, became St. Thomas's first treasurer.

The Founding of Guy's Hospital.

There was a record of steady progress at the hospital, but again, at the end of the seventeenth century, the buildings had got into a bad state of repair, so that it became necessary to rebuild. One of the greatest figures in the history of the hospital, Sir Robert Clayton, undertook this rebuilding, and three squares were erected in Southwark in 1695, and were completed in the early part of the eighteenth century. One of the wings in the front quadrangle was erected at the sole expense of Thomas Guy. It was now felt that the hospital had sufficient for its purpose, and, though Guy was anxious to do more, it was thought that his wealth should be devoted to another object. He then enlisted the assistance, and was guided by the judgment, of Richard Mead, one of the most notable physicians in the history of St. Thomas's. Some land was secured on a long lease from the Governors of St. Thomas's, and Guy's Hospital was erected for the reception of cases who needed longer treatment than could be afforded by St. Thomas's at that time. For many years these two institutions worked side by side, their medical schools forming one whole, the surgical teaching being mostly done by the St. Thomas's men, the medical by the Guy's teachers.

The Hospital Sold for £296,000.

Mr. Roberts then recited the changes involved in 1859 by the building of London Bridge, which necessitated the reconstruction of the front part of the hospital and meant interfering with the northern block of St. Thomas's, which had been completed in 1848. The Governors urged that if the railway company wanted any land at all they must take the whole. The usual litigation ensued, and Vice-Chancellor Wood decided that under the Land Clauses Consolidation Act the view taken by the hospital Governors was the correct one, and the railway company would be obliged to purchase the whole hospital. In 1861, Mr. John Stewart, of Liverpool, was appointed umpire of the arbitration. The hospital claimed £478,000; the company offered £145,000. Mr. Stewart awarded £296,000, and in 1862 the company took possession of the hospital premises. Temporary accommodation for the patients was secured in the Surrey Gardens, which provided 200 beds. Ultimately the surplus land created by the erection of the Albert Embankment was decided upon, and 8½ acres were purchased for £90,000 from the Metropolitan Board of Works.

The Present St. Thomas's.

The designs for the new hospital were completed in 1865 to the satisfaction of the Governors, the architect having been Henry Curry. Tenders for its erection were received in July 1867. That of John Perry and Company was accepted, for the sum of £332,000. The foundation stone was laid on May 13, 1868, by Her Majesty Queen Victoria, in the presence of 3,000 spectators. The twenty-five million bricks were brought from Fareham, Hants, by boat to the Embankment, and the ironwork, 1,250 tons, came from Belgium. The cost of the building, exclusive of site, was over £400,000, or £650 per bed.

The remainder of the lecture and pictures was devoted to showing the details of the work now being carried on by the hospital. Special attention was drawn to the great improvements which had been carried out in the building during the past twenty years. The hospital had always been ready to carry out any new treatment required, and it was the first of the large hospitals to establish a tuberculosis dispensary. With regard to the establishment of a special maternity department for the admission of difficult cases of labour, the Governors took a notable step by approaching the general practitioners in the neighbourhood, and co-operating with them as to the cases to be admitted. A department for the treatment of venereal disease had already been established before the present national provision, and it had been brought up to date. The necessity for social education among the people was emphasised by photographs of homes in some of the poorer streets of Lambeth, and in this connection Mr. Roberts spoke of the hospital's fine social work under the direction of the Lady Almoner, and the work in furtherance of baby welfare.

At the beginning of the war the hospital offered accommodation for soldiers, which the Government readily accepted. Huts were erected in the hospital squares, and the Fifth London General Hospital constituted, with 530 beds. The entertainment side had been most assiduously and generously looked after by Mrs. Herbert and Miss Ismay.

